# Local advanced adrenocortical cancer with a long‐term recurrence‐free survival treated with complete surgical excision and adjuvant therapy with a very low‐dose mitotane

**DOI:** 10.1002/iju5.12196

**Published:** 2020-07-15

**Authors:** Yuumi Tokura, Minoru Kobayashi, Takao Kamai

**Affiliations:** ^1^ Department of Urology Dokkyo Medical University School of Medicine Tochigi Japan; ^2^ Department of Urology Utsunomiya Memorial Hospital Tochigi Japan

**Keywords:** adrenocortical cancer, long‐term survival, mitotane

## Abstract

**Introduction:**

Adrenocortical cancer is a rare tumor with high malignant potential, often resulting in recurrence and poor survival even after complete excision. Thus, adjuvant therapy needs to be considered despite its unestablished effectiveness.

**Case presentation:**

We report a 70‐year‐old man presented with a huge adrenal mass extending into the right liver lobe. He underwent right adrenalectomy concurrent with right nephrectomy and right hemihepatectomy for curative intent. He was given a very low‐dose mitotane (0.5 g/day) adjuvant therapy to be alive without any signs of recurrence for 90 months after surgery without unwanted toxicity.

**Conclusion:**

A long‐term and very low dose of adjuvant mitotane could be an option for a patient with complete surgical resection in an expectation to prevent microscopic residual disease from recurrence.

**Abbreviations & Acronyms**
ACC: adrenocortical cancer
CT: computed tomography
ENSAT: European Network for the Study of Adrenal Tumors
MRI: magnetic resonance imaging
PET: positron emission tomography
RFS: recurrence‐free survival
TDM: therapeutic drug monitoring

**Keynote message**

ACC is a rare tumor with high malignant potential and a poor prognosis even after successful surgery. A long‐term and very low dose (i.e. 0.5 g/day) of adjuvant mitotane could be an option for a patient with complete surgical resection to prevent microscopic residual disease from recurrence without toxicity.

## Introduction

ACC is a rare tumor with high malignant potential. The patient of ACC often experience recurrence and difficulty to achieve long‐term survival even after successful surgery. Thus, adjuvant therapy needs to be considered for patients without macroscopic residual tumor after surgery but who may potentially have the likelihood of recurrence. Here, we report a case of long‐term survivor of local advanced ACC treated with a very low‐dose mitotane after complete excision.

## Case presentation

**Fig. 1:**
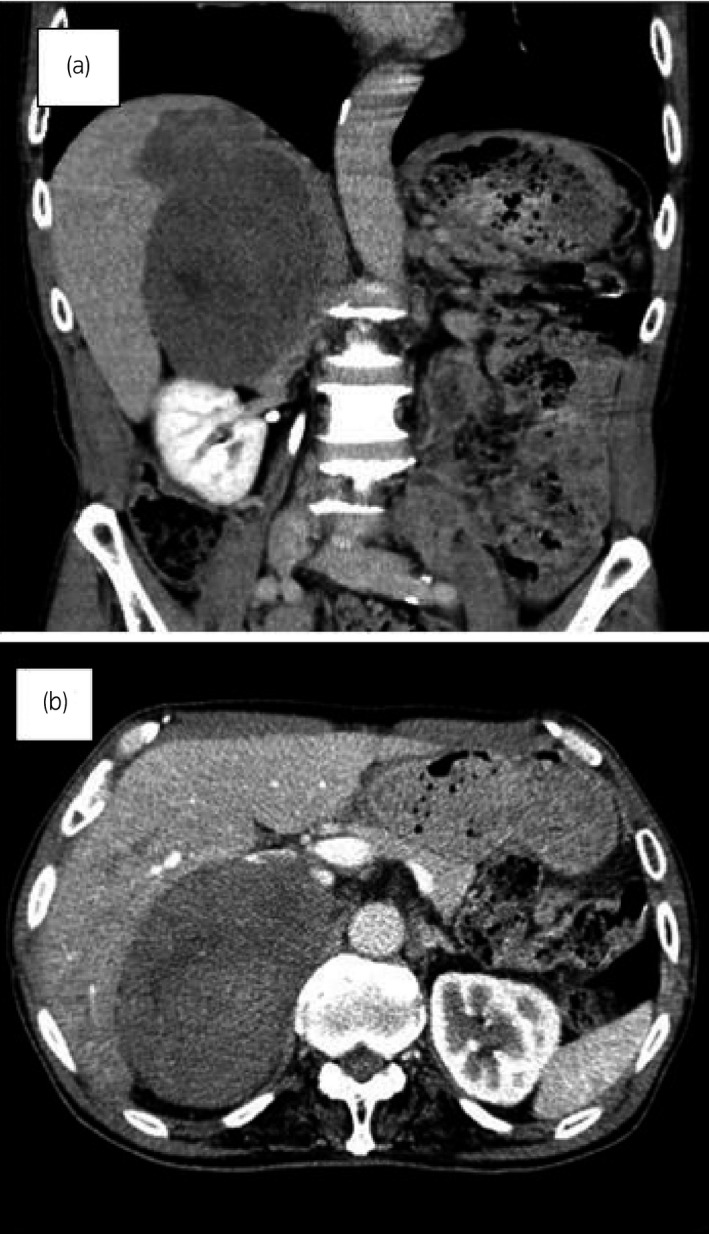
CT of the abdomen (a: coronal image, b: axial image). A huge adrenal mass with a maximum diameter of 15 cm which extended to the right liver lobe.

**Fig. 2:**
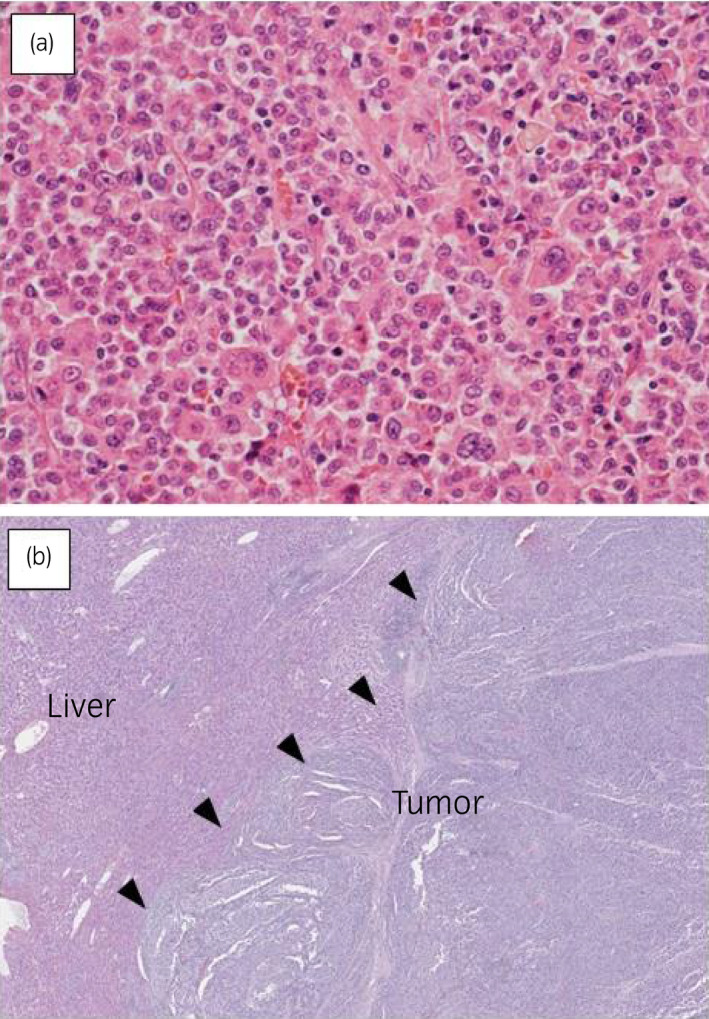
(a) Histopathological specimen of the adrenal tumor (HE staining, original magnification: ×400). This case was scored five of nine items in Weiss criteria: high nuclear grade (Fuhrman G4), one mitotic figure/3 HPF, eosinophilic cells, diffuse architecture in almost all tumors, and microscopic necrosis. (b) Histopathological specimen of the adrenal tumor infiltrating to the liver (HE staining, original magnification: ×20). Arrowheads indicate the boundary between the tumor and the liver, where tumor capsule is not identified.

A 70‐year‐old man presented with right back pain and appetite loss to our hospital. Abdominal CT revealed a huge adrenal mass with a maximum diameter of 15 cm which extended to the right liver lobe (Fig. 1). The tumor showed heterogeneous density with uneven enhancement, reflecting intra‐tumor necrosis and hemorrhage. The features of the tumor on MRI were low intensity on T1‐weighted image and high intensity on T2‐weighted image. PET showed an intense fluorodeoxyglucose uptake in the right liver lobe (SUVmax 17.5), suggestive of tumor invasion. Vena cavography suggested tumor compression rather than direct invasion to the inferior vena cava wall. General laboratory testing data were normal range. Endocrine examination with respect to the hypothalamic‐adrenal axis is shown in Table 1. The mild elevation of noradrenaline level may not be specific since the level was not lowered but increased after operation and following mitotane administration. Taken these findings together, his clinical diagnosis was ACC, cT4N0M0, UICC/WHO Stage IV. Thus, right adrenalectomy concurrent with right hemihepatectomy as well as right nephrectomy were performed. Operation time was 6 h and 40 min and blood loss was 1880 mL. Histologically, the tumor cells showed diffuse cytoplasmic eosinophilia and nuclear polymorphism such as enlarged, bizarre nucleus, multiple nuclei, and atypical mitotic figure (Fig. 2a). The tumor exclusively infiltrated into the liver to form a mass lacking a tumor capsule (Fig. 2b). The histopathological diagnosis was ACC, RM0, five score in Weiss criteria (Table 2),^1^ leading to the final diagnosis of ACC, pT4N0M0, ENSAT Stage III. The postoperative course was uneventful. The patient received a very low‐dose mitotane with an initial dose of 0.5 g/day at a month after surgery to be maintained at the same dose without any adverse events. He is alive with no sign of recurrence at 90 months after surgery.

**Table 1:** Hormonal examination during the treatment course

	Before operation	Before mitotane	After mitotane
Adrenaline pg/mL (<100)	41	39	40
Noradrenaline pg/mL (100–450)	639	1373	1000
Dopamine pg/mL (<20)	14	38	26
11‐hydroxycorticosteroid μg/dL (0.4–11.6)	11.2	–	–
11‐OH‐androsterone mg/day (0.4–2.3)	–	0.61	0.74
Aldosterone pg/mL (57–150)	60.7	48.3	90.9
Urinary homovanillic acid mg/day (2.1–6.3)	7	2.6	2.8
DHEA‐S μg/dL (5–253)	426	–	–
DHEA mg/day (0.12–5.2)	–	0.1	0.06

**Table 2:** Histopathologic criteria by Weiss

High nuclear grade
Mitotic rate greater than 5 per 50 high power fields (HPF)
Atypical mitotic figures
Eosinophilic tumor cell cytoplasm (greater than 75% tumor cells)
Diffuse architecture (greater than 33% of tumor)
Necrosis
Venous invasion
Sinusoidal invasion
Capsular invasion

## Discussion

ACC is a rare tumor with high malignant potential. The patient of an advanced stage of ACC often experience recurrence and difficulty to achieve long‐term survival even after complete excision.^2^ Based on the ENSAT staging system, 5‐year survival rates are 82% in Stage I, 61% in Stage II, 50% in Stage III, and 13% in Stage IV.^3^

Thus, even in nonmetastatic disease, adjuvant treatment is recommended due to high recurrence rate.^4^ Mitotane (o,p’‐dichlorodiphenyldichloroethane) is the only approved drug for treatment of ACC. This drug causes selective cytotoxic atrophy of the adrenal cortical cells, but the definitive mechanism of antitumor effect has not been clarified. Although no randomized clinical trials have been conducted to explore its efficacy as adjuvant setting, six studies evaluated the effect of mitotane on recurrence and mortality,^5^, ^6^, ^7^, ^8^, ^9^, ^10^ four of which favored mitotane treatment over no treatment. In the most recent retrospective case‐control study by Berruti *et al.*,^10^ 162 ACC patients who underwent radical surgery were divided into three groups: 47 patients received adjuvant mitotane, 45 patients (control group 1) and 70 patients (control group 2) without adjuvant treatment. Median RFS was 42 months in the mitotane group, 17 months in control group 1, and 26 months in control group 2, respectively. Median duration of adjuvant treatment was 42 months (range 4–162), doses ranging daily from 3 to 20 g. The survival benefit of adjuvant mitotane was confirmed regardless of the hormone secretary status.^10^

As the effective therapeutic range of mitotane is narrow because of its toxicity,^11^ TDM is necessary. It has been shown that mitotane blood concentration between 14 and 20 mg/L is associated with therapeutic efficacy in balance with toxicity in ACC.^12^, ^13^ It is recommended that mitotane should be administered in an escalating manner depending on the patient’s performance status and the tolerability at initial phase of treatment. A high‐dose regimen with a starting dose of 1.5 g/day and dose escalation by 1.5 g up to 6 g/day was proposed to allow a rapid rise of blood concentration despite the concerned toxicity. On the other hand, a low‐dose regimen with a starting dose of 1 g/day and dose escalation by 0.5 g up to 3~4 g/day is also able to achieve target concentration with better tolerability, but with a delay of several months from treatment start.^13^ The optimum duration of mitotane treatment is not defined, but one guideline suggests to administer adjuvant mitotane for at least 2 years, but not longer than 5 years.^2^ In Europe, mitotane monitoring is readily available as a free service provided by the company distributing mitotane (info@lysodren‐europe.com). However, the measurement of mitotane concentration is not covered by medical insurance in Japan, and this is a barrier to optimal practice for mitotane treatment. Therefore, we decided to start mitotane adjuvant treatment at a very low dose of 0.5 g/day to be maintained at the same dose, which was much less than the recommended dose in the previous reports. The patient did not have any adverse events even without corticosteroid replacement, suggesting that the blood concentration should be far below the effective level. Nevertheless, the patient achieved a long‐term RFS of 7.5 years. This implies that only complete resection (RM0) might contribute to long‐term RFS, otherwise even very low dose of mitotane may be enough to suppress microscopic residual tumor cells to emerge as clinical recurrence when administered for a prolonged period of time.

In conclusion, ACC is a rare tumor with high malignant potential and a grim prognosis often resulting in recurrence and poor survival even after successful surgery. A long‐term and very low dose of adjuvant mitotane (i.e. 0.5 g/day) could be an option for a patient with high risk of recurrence despite complete surgical resection, when plasma concentration monitoring is unavailable, in an expectation to prevent microscopic residual disease from recurrence without unwanted toxicity.

## Conflict of interest

The authors declare no conflict of interest.
